# Adenosine for postoperative analgesia: A systematic review and meta-analysis

**DOI:** 10.1371/journal.pone.0173518

**Published:** 2017-03-23

**Authors:** Xin Jin, Weidong Mi

**Affiliations:** Department of Anesthesiology, Chinese PLA General Hospital, Beijing, China; Nanjing University Medical School Affiliated Nanjing Drum Tower Hospital, CHINA

## Abstract

**Purpose:**

Perioperative infusion of adenosine has been suggested to reduce the requirement for inhalation anesthetics, without causing serious adverse effects in humans. We conducted a meta-analysis of randomized controlled trials evaluating the effect of adenosine on postoperative analgesia.

**Methods:**

We retrieved articles in computerized searches of Scopus, Web of Science, PubMed, EMBASE, and Cochrane Library databases, up to July 2016. We used adenosine, postoperative analgesia, and postoperative pain(s) as key words, with humans, RCT, and CCT as filters. Data of eligible studies were extracted, which included pain scores, cumulative opioid consumption, adverse reactions, and vital signs. Overall incidence rates, relative risk (RR), and 95% confidence intervals (CI) were calculated employing fixed-effects or random-effects models, depending on the heterogeneity of the included trials.

**Results:**

In total, 757 patients from 9 studies were included. The overall effect of adenosine on postoperative VAS/VRS scores and postoperative opioid consumption was not significantly different from that of controls (P >0.1). The occurrence of PONV and pruritus was not statistically significantly different between an adenosine and nonremifentanil subgroup (P >0.1), but the rate of PONV occurrence was greater in the remifentanil subgroup (P <0.01). Time to first postoperative analgesic requirement in the adenosine group was not significantly difference from that of the saline group (SMD = 0.07, 95%CI: −0.28 to 0.41, P = 0.71); but this occurred significantly later than with remifentanil (SMD = 1.10, 95%CI: 2.48 to 4.06, P < 0.01). Time to hospital discharge was not significantly different between the control and adenosine groups (P = 0.78). The perioperative systolic blood pressure was significantly lower in the adenosine than in the control group in the mannitol subgroup (P < 0.01). The incidence of bradycardia, transient first- degree atrioventricular block, and tachycardia was not significantly different between the adenosine and control groups (P > 0.1).

**Conclusion:**

Adenosine has no analgesic effect or prophylactic effect against PONV, but reduce systolic blood pressure and heart rates. Adenosine may benefit patients with hypertension, ischemic heart disease, and tachyarrhythmia, thereby improving cardiac function.

## Introduction

Postoperative pain can have a significant effect on patient recovery. The results of 1 survey have indicated that about 80% of patients who undergo surgery experience severe postoperative pain[1].Preemptive analgesia has therefore been widely used in the clinical setting; opioids remain the mainstay for postoperative analgesia, especially after major surgery[2]. However, opioid- related side-effects can be distressing to patients, and include respiratory depression, post-operative nausea and vomiting (PONV), urinary retention, sedation, and pruritus[3].To improve analgesic quality, adjunct medications are used; such multimodal analgesia allows more optimal pain management[4].

Adenosine(ADO) is a breakdown product of adenosine triphosphate(ATP); it is an endogenous purine nucleoside with several biological effects. It is involved in numerous biological processes, including neurotransmission, muscle contraction, heart function, and has anti-inflammatory activity[5]. Perioperative infusion of low-dose adenosine in recent studies has been found to reduce the requirements for inhalation anesthetics[6]. It could be argued that the vasodilatory effect of ADO has a major influence on the stability of systolic blood pressure (SBP) during surgery. 2 groups with similar presurgical ET-IS0 concentrations indicated the absence of significant ADO-induced reduction of SBP. This further implies that the maintained SBP reported in the ADO group during surgery is unrelated to an ADO-mediated vascular effect, but is rather due to an ADO-mediated modulation of the afferent reflex response to surgical trauma, which is related to the antinociceptive action of adenosine [6]To date, no serious adverse effects of ADO treatment have been reported in humans[6,7].

Here, we performed a meta-analysis to evaluate the impact of perioperative administration of ADO on postoperative pain, PONV, and the cardiovascular system.

## Methods

We followed the recommendations of the PRISMA statement[8]

### Search strategy

Published reports [2,6,7,9–16,] in English were retrieved in a computerized search of Scopus, Web of Science, PubMed, EMBASE, and Cochrane Library databases, for the period up to July 2016. The keywords used to search these databases were adenosine, postoperative analgesia and postoperative pain(s), and humans, RCT, and CCT were used as filters. We also examined similar published research with retrieved literatures.

### Study selection

**Fig 1:**
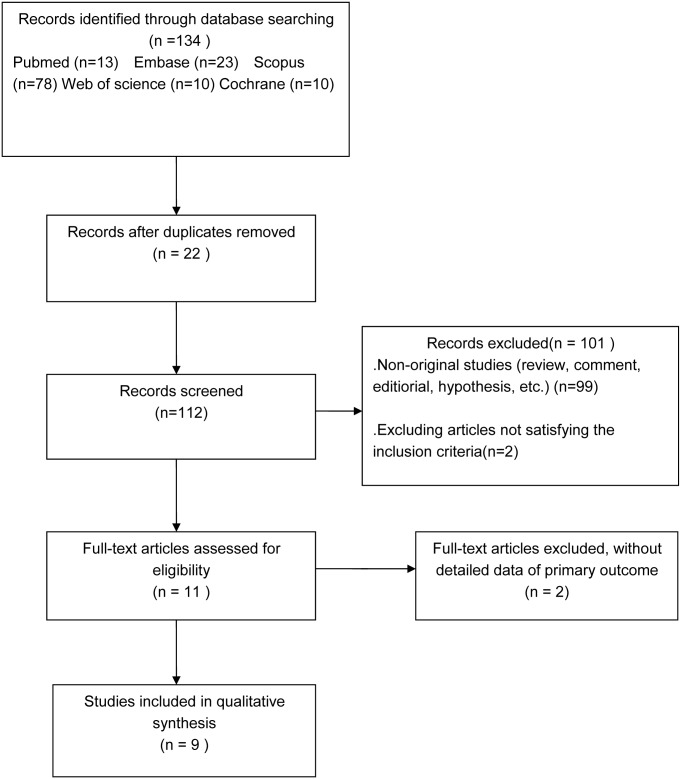
Flow chart of selection of studies for inclusion in the present meta-analysis.

The details of the study selection processes are shown in Fig 1. The database search yielded 134 potentially relevant papers. However, 121 studies were excluded after reviewing the title and abstract as these included duplicated and non-original studies, while two were excluded as they did not meet the inclusion criteria; 11 studies were thus further investigated [2,6,7,9–16]. A total of 448 patients who received ADO infusions and 403 controls were enrolled [2,6,7,9–16]. However, there were 2 studies in which the data were presented as bar graphs, and despite having contacted the corresponding authors, no further information could be obtained[10,11]. Therefore, eventually, only 9 studies were included [2,6,7,9, 12–16].

### Data selection and extraction

#### Inclusion criteria

The criteria for inclusion of papers were as follows: (1) randomized assignment of patients to treatment groups, (2)double-blind assessments of pain and analgesic use, (3) report of pain using a reliable and valid measure, (4)report of analgesic consumption, (5)absence of obvious methodological problems.

The primary outcomes included postoperative pain scores and postoperative cumulative opioid consumption at 4h, 24 h, and 48h. Secondary outcomes included pain scores and opioid consumption at 4h, 24 h and, 48h, and adverse reactions (PONV and pruritus) at 1–24 h. Tertiary outcomes included patients’ heart rate, blood pressure early, mid, and late during the surgical period. If results were not reported at the exact time-points specified above, those recorded closest to that time-point were used instead [17].

The eligible literature reports were assessed by 2 investigators, independently, according to the Cochrane Handbook guidelines. They extracted the following data: (1) surgical procedure, (2) mode,dose, and time-point of ADO administration, (3) number and gender of subjects, (4)American Society of Anesthesiologists grade, (5) primary outcome of the studies, (6) pain scores (rest/movement), (7) opioid consumption, and (8) adverse reactions (nausea, vomiting, pruritus, and sedation).If the data were presented as graphs/charts,the authors were contacted to provide specific data.

#### Definition of outcomes

Pain was rated using avisual analogue scale(VAS, range 0–10) or verbal/ numerical rating scale(VRS, range 0–100). We used the continuous data to obtain 95% confidence intervals(CIs) and the standardized mean differences (SMDs).If the 95% CIs included ‘‘0”,it was implied that there was no statistically significance difference between treatment and control groups. We used dichotomous data to obtain relative risks (RRs); if the 95% CIs included “1,”it also implied a lack of statistically significant difference between groups.

### Data synthesis and analysis

All the analyses were conducted using Review Manager version 5.3 (Nordic Cochrane Centre, Copenhagen, Denmark).Heterogeneity was analyzed using the chi-square test. If P>0.1, which implied the absence of statistical heterogeneity among these studies, the fixed-effect (FE) model was applied to the meta-analysis. If P ≤ 0.1, I^2^ was assessed. If I^2^ > 50%, which implied significant heterogeneity, the random-effect (RE) model was applied to the meta-analysis. Sensitivity analysis and subgroup analysis were based on the primary outcome data. Publication bias was assessed by funnel plot and Egger test. We defined statistical significance by a 2-sided P < 0.05.

## Results

### Descriptions of the included randomized controlled trials

Four studies were conducted in Sweden[6,7,9,10], 2 studies in the USA [2,14], 2 studies in Turkey[11,12], 2 studies in India[13,16], and 1 study in Korea[15].6 studies included only female participants[2,7,9,10,13,16], while 5 studies included both male and female subjects [5,6,11,12,15].7 studies administered ADO via an intravenous route[2,6,7,9,12,14,15], and the remaining 4 studies did so via the intrathecal route [10,12,16]. The characteristics of these studies are summarized in Table 1, and risk of bias in these studies is summarized in Table 2.

**Table 1:** Characteristics of included studies (n = 757).

First author/year	Age (ys)	F/M	Number of patients (case/control)	ASA	ADO dose	Surgery	Anesthesia method	ADO route	Primary outcome	second outcome	Intra-operative analgesia	PostoperativeAnalgesia	Case/Control
**Segerdahl M./1995**	18–70	70/0	70(36/34)	I–II	80 pg·kg^-1^·min^-1^	Breast surgery	GA	iv	Pain scores	opioid consumption	O2/N2O/ISO +ALF	MOR/CET	MAN+ADO/ MAN
**Segerdahl M./1996**	19–62	7/23	30(14/16)	I–II	80 pg·kg^-1^·min^-1^	Shoulder joint surgery	GA	iv	Pain scores	PONV(nausea)	O2/N2O/ISO	MOR iv/im	MAN+ADO/ MAN
**Segerdahl M./1997**	32–65	43/0	43(23/20)	I–II	80 pg·kg^-1^·min^-1^	Abdominal hysterectomy	GA	iv	Pain scores	opioid consumption	O2/N2O/ ISO +ALF/FEN	MOR/KET iv	MAN+ADO/ MAN
**Rane K./2000**	37–66	42/0	42(21/21)	I–II	500 μg	Abdominal hysterectomy	GA	it	Pain scores	opioid consumption	O2/N2O/ISO+FEN	CET by PCA /after48h by OA	ADO/SA
**Apan A./2003**	25–55	38/12	50(25/25)	I–II	80μg·kg^-1^·min^-1^	Upper limb surgery	SA	it	Time to first complaint of pain	analgesic requirement	PRL/LGN	DIC	ADO/ SA
**Apan A./2003**	25–55	44/18	60(30/30)	I–II	80 μg·kg^-1·^min^-1^	Upper extremity surgery	BPB	iv	Pain scores	opioid consumption	LD	MET iv	ADO/SA
**Ghai A./2011**	40–60	75/0	75(50/25)	I–II	500 μg/1000 μg	Vaginal hysterectomy	SA	it	Sedation scores	Pain scores	FEN	FEN by PCA	ADO/SA
**Sharma M./2006**	30–60	90/0	90(60/30)	I–II	1000 μg	Abdominal hysterectomy	GA	it	Pain scores	Time to first rescue analgesia	O2/N2O/HL+MOR	MOR iv	Early(late) ADO/SA
**FukunagaA.F./2003**	35–60	33/8	41(20/21)	I–II	50–500 μg·kg^-1^·min^-1^	Total abdominal hysterectom/total knee arthroplasty/total hip arthroplasty	GA	iv	Pain scores	Sedation scores	ADO/REM	MOR by PCA /FEN iv	ADO/REM
**Habib A. S./2008**	18–70	166/0	166(125/41)	I–III	25, 50, 100, 200 μg·kg^-1^·min^-1^	Major gynecologic surgery	GA	iv	Pain scores	opioid consumption	O2/N2O/ISO+FEN	MOR/KET by PCA	ADO/SA
**Lee C./2011**	30–50	39/51	90(30/60)	I–II	80 μg·kg^-1^·min^-1^	Tonsillectomy	GA	iv	Pain scores	opioid consumption	REM/SEV	PET/ KTR	ADO/REM/ SEV

Ys = years, F/M = females/male, ASA = American Society of Anesthesiologists,ADO = adenosine, GA = general anesthesia, SA = subarachnoid/ spinal anesthesia, BPB = Brachial plexus block, iv = intravenous injection; it = intrathecal injection; im = intramuscular injection, PCA = patient-controlled analgesia, ISO = isoflurane, ALF = alfentanil, FEN = fentanil, PRL = prilocaine, LGN = lignocaine, LD = Lidocaine, HL = halothane, MOR = morphine, REM = remifentanil,SEV = sevoflurane, CET = cetobemidone, KET = ketobemidone, OA = oral analgesics, DIC = diclofenac, MET = metamizol, PET = pethidine,KTR = ketorolac, MAN = Isotonic mannitol,SA = saline

**Table 2:** Risk of bias.

First author/year	Adequate sequence generation	Allocation concealment	Blinding of participants and personnel	Blinding of outcome assessment	Incomplete outcome data	Selective reporting
**Segerdahl M./1995**	LOW	LOW	LOW	LOW	LOW	LOW
**Segerdahl M./1996**	HIGH	LOW	LOW	LOW	LOW	LOW
**Segerdahl M./1997**	HIGH	LOW	LOW	UNCLEAR	LOW	LOW
**Rane K./2000**	LOW	LOW	LOW	LOW	LOW	LOW
**Apan A./2003**	LOW	LOW	HIGH	HIGH	LOW	LOW
**Apan A./2003**	LOW	LOW	LOW	LOW	LOW	LOW
**Ghai A./2011**	LOW	LOW	LOW	LOW	LOW	LOW
**Sharma M./ 2006**	LOW	HIGH	HIGH	HIGH	LOW	LOW
**FukunagaA.F./ 2003**	HIGH	HIGH	LOW	LOW	UNCLEAR	LOW
**Habib A. S./2008**	LOW	LOW	LOW	UNCLEAR	LOW	LOW
**Lee C./2011**	LOW	HIGH	HIGH	LOW	LOW	LOW

Low = low risk of bias; unclear = unclear risk of bias; high = high risk of bias.

### Primary outcomes

#### Pain scores at 4 h after surgery

**Fig 2:**
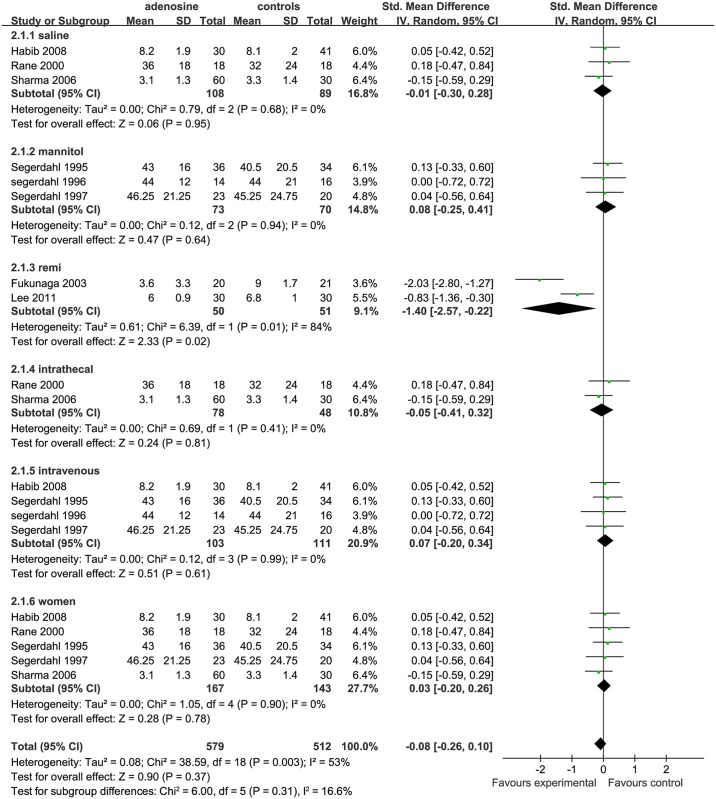
Forest plot of pain scores at 4 h after surgery. SD = standard deviation; CI = confidence interval; MD = mean difference; W = weight; remi = remifentanil subgroups; I^2^ = heterogeneity.

**Fig 3:**
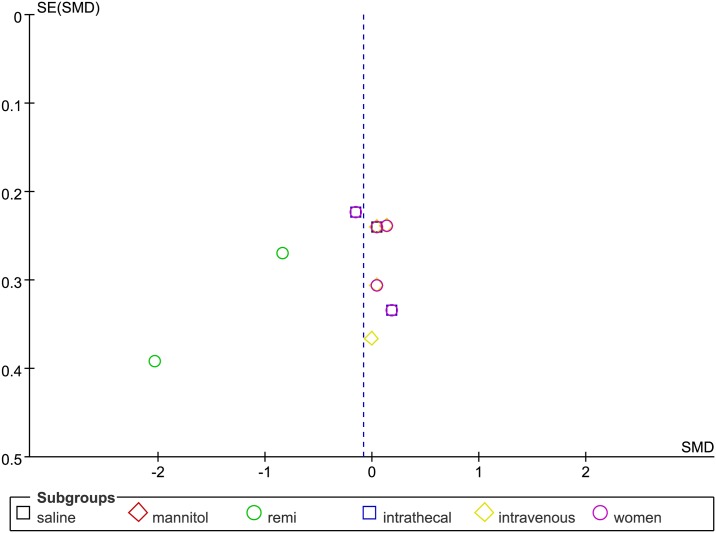
Funnel plot of effect on pain scores at 4 h after surgery. SE = standard error; SMD = Standardized Mean Difference.

The overall effect of ADO on pain scores at 4 h after surgery, as compared with controls, showed no significant difference (SMD = -0.08, 95%CI: -0.26 to 0.10, I^2^ = 53%, P = 0.37),according to the forest plot (Fig 2). The funnel plot showed significant asymmetry (P < 0.05; Fig 3).

*Drug categories in control group analysis* In terms of the control group,3 studies used saline as placebo(saline subgroup) [2, 10, 16], 3 studies used ADO+mannitol in the treatment group and used only mannitol in the control group (mannitol subgroup)[6,7,9], and 2 studies used remifentanil as control (remifentanil subgroup) [14,15]. Compared with the ADO group, pain scores at 4 h after surgery in the saline (SMD = −0.01, 95%CI: −0.30 to 0.28, I^2^ = 0%, P = 0.95)and mannitol (SMD = 0.08, 95%CI: −0.25 to 0.41,I^2^ = 0%, P = 0.64) subgroups were similar; but the pain scores at 4 h after surgery in the remifentanil subgroup were higher (SMD = −1.40, 95%CI: −2.57 to −0.22, I^2^ = 84%, P = 0.02). The analysis showed marked heterogeneity (I^2^ = 84%,Fig 2).

*Drug- delivery route analysis* Four studies administered ADO by intravenous injection (IV subgroup) [2,6,7,9], 3 studies did so by intrathecal injection (IT subgroup) [10,16], and 2 studies used remifentanil as control(remifentanil subgroup) [14,15].It was not possible to combine this group with the other studies, as they showed statistically significant heterogeneity. Compared with the ADO group, the pain scores at 4 h after surgery in the IV subgroup (SMD = 0.07, 95%CI: −0.20 to 0.34, I^2^ = 0%, P = 0.61) and IT subgroup (SMD = −0.05, 95%CI: −0.41 to 0.32, I^2^ = 0%, P = 0.81) were similar (Fig 2).

*Sex subgroup analysis* Six studies included only female patients(women subgroup) [2,6,7,9,11,13].As the study by Ghai et al.[13]did not include the standard deviation for VAS pain scoring, it had to be excluded for the sake of accuracy. Compared with the ADO group, *Sex* in the women subgroup (SMD = −0.03, 95%CI: −0.20 to 0.26, I^2^ = 0%, P = 0.78) was not statistically significantly different from a combined man/women subgroup (Fig 2).

#### Pain scores at 24 h after surgery

**Fig 4:**
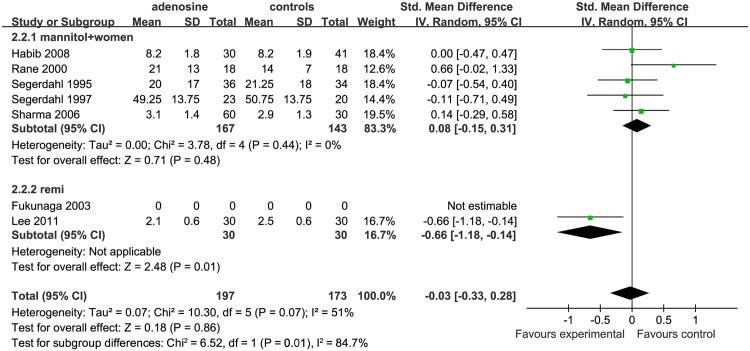
Forest plot of pain scores at 24 h after surgery. SD = standard deviation; CI = confidence interval; MD = mean difference; W = weight; remi = remifentanil subgroups; I^2^ = heterogeneity.

According to the overall forest plot for the effect of ADO on pain scores at 24 h after surgery, VAS/VRS scores in the treatment group was not significantly different from that in the control group (SMD = −0.03, 95%CI: −0.33 to 0.28, I^2^ = 51%,P = 0.86; Fig 4). As only 6 studies investigated this time-point, the funnel plot was not suitable for evaluating publication bias.

Three studies used saline in the controls(saline subgroup) [2,10,16], two studies used ADO with mannitol in the treatment and mannitol in the control group (mannitol subgroup) [6,7,9]. Overall, these 5 studies were included in the non-remifentanil subgroup (nonremi).Two studies used remifentanil in the controls(remifentanil subgroup) [14,15].

Compared with the ADO group, VAS/VRS scores at 24 h after surgery in the nonremi (SMD = 0.08, 95%CI −0.15 to 0.31, I^2^ = 0%, P = 0.48) subgroup were not statistically significantly different between the ADO group and nonremi subgroup; however, these scores were higher in the remifentanil subgroup (SMD = −0.66, 95%CI: −0.18 to −0.14, P = 0.01).

#### Pain scores at 48 h after surgery

Only 1 study[2]examined the pain scores at 48 h after surgery s. In that study, there was no significant difference between the control group (a saline group) and the ADO group (P = 0.14).

#### Cumulative postoperative opioid consumption

**Fig 5:**
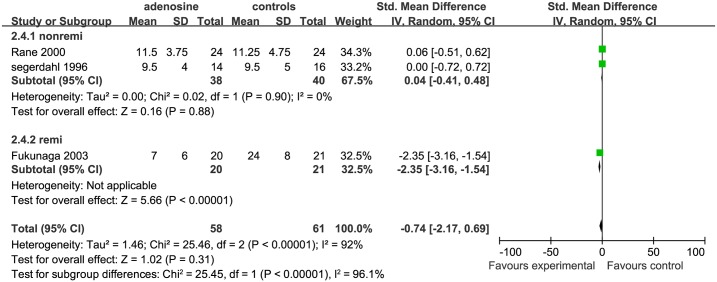
Forest plot of cumulative opioid consumption at 4 h postoperatively. H = hour; SD = standard deviation; CI = confidence interval; MD = mean difference; W = weight; remi = remifentanil subgroups; nonremi = subgroups without remifentanil administration; I^2^ = heterogeneity.

The overall effect of ADO on early pain in terms of postoperative opioid consumption was not significantly different from that of control treatments (SMD = −0.74, 95%CI: −2.17 to 0.69,I^2^ = 92%, P = 0.31) according to the forest plot (Fig 5). As this was recorded by only 6 studies, the funnel plot was not suitable for evaluating publication bias.

*Cumulative opioid consumption at 4h postoperatively* In terms of cumulative opioid consumption at 4 h, 3 studies were included: 2 for the nonremi subgroup [6,10] and 1 for the remifentanil subgroup [14].We show the cumulative opioid consumption at 4 h separately for these subgroups. In the nonremi subgroup, outcomes of the control groups were not significantly different from those of the ADO group (SMD = 0.04, 95%CI: −0.41 to 0.48, I^2^ = 0%, P = 0.88); in the remifentanil subgroup, the control group used significantly more opioids than did the ADO group (SMD = −2.35, 95%CI: −3.16 to −1.54, P < 0.00001).

**Fig 6:**
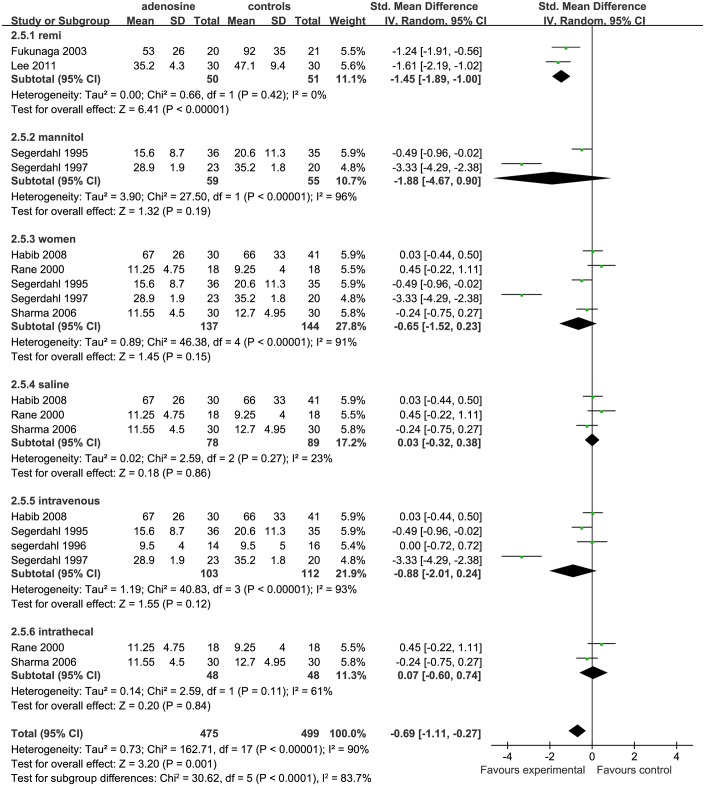
Forest plot of cumulative opioid consumption at 24 h postoperatively. H = hour; SD = standard deviation; CI = confidence interval; MD = mean difference; W = weight; remi = remifentanil subgroups; nonremi = subgroups without remifentanil administration; I^2^ = heterogeneity.

**Fig 7:**
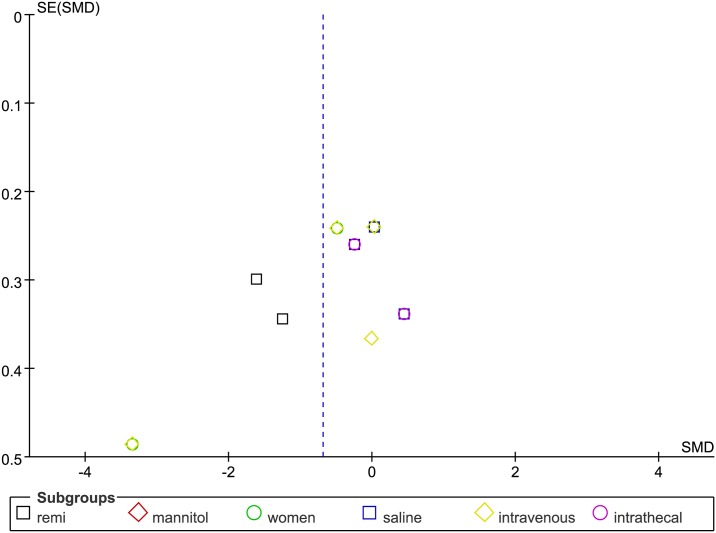
Funnel plot of cumulative opioid consumption at 24 h postoperatively. SE = standard error; SMD = Standardized Mean Difference.

*Cumulative opioid consumption at 24h postoperatively* The overall forest plot of cumulative opioid consumption at 24h showed that this value was statistically significantly higher for controls than for the ADO treatment group (SMD = −0.69, 95%CI-1.11 to −0.27, I^2^ = 90%, P = 0.001; Fig 6).The funnel plot showed significant asymmetry (P < 0.05; Fig 7).

According to all impact factors, as previously stated, 3 studies were included in a saline subgroup [2,10,16];2 studies were included in a mannitol subgroup [7, 9];2 studies were included in the remifentanil subgroup [13,14]4 studies were included in the IV subgroup [2,6,7,9]; 3 studies were include in the IT subgroup[10,16]; and 6 studies were included in the women subgroup [2,6,7,9, 10,13]. Compared with ADO group, cumulative opioid consumption at 24h in the saline (P = 0.86), IT (P = 0.84), mannitol(P = 0.19), and women (P = 0.15) subgroups was not statistically significantly different between the treated subjects and controls; Nevertheless,in the remifentanil subgroup, the control group was administrated more opioid than the ADO treatment group (SMD = −1.45, 95%CI: −1.89 to −1.00, I^2^ = 0%, P <0.00001).

**Fig 8:**
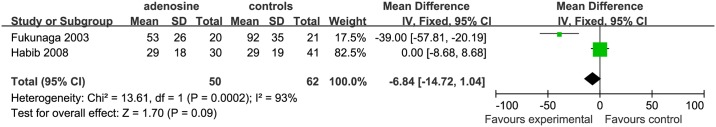
Forest plot of cumulative opioid consumption at 48 h postoperatively. H = h; SD = standard deviation; CI = confidence interval; MD = mean difference; I^2^ = heterogeneity.

*Cumulativeopioid consumption at 48h postoperatively* Two studies [2, 14] were included in the analysis of the cumulative opioid consumption at 48h. This value was significantly more in the control than in the ADO group (SMD = −6.84, 95%CI: −14.72 to1.04, I^2^ = 93%, P < 0.09, Fig 8). The heterogeneity in the data was higher, as one study [2] used saline in the controls (SMD = 0, 95%CI: −8.68 to 8.68); but the other study [17] used remifentanil in the controls (SMD = −39.00, 95%CI: −57.81 to −20.19). In the study by Fukunaga et al. [14], the usage of cumulative opioid consumption at 48 h was less in the ADO group than in the controls.

### Secondary outcomes

#### Postoperative nausea and vomiting

Four studies examined the effect of ADO on PONV [2, 6,13, 14]. In 4 of these studies studies, except for that by Ghai et al. [13], nausea was combined with vomiting as a single outcome, while the other studies [2, 8, 14] considered them as separate outcomes.

**Fig 9:**
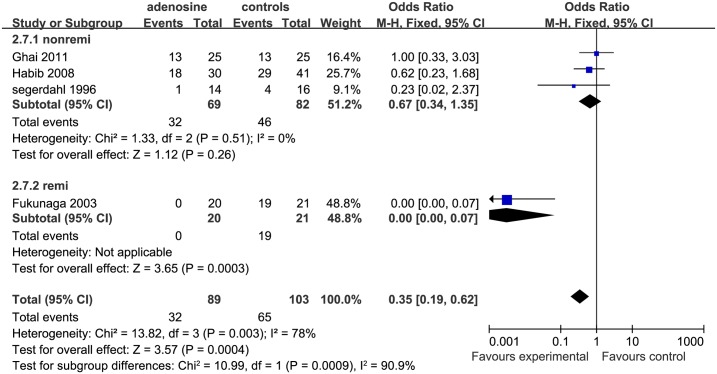
Forest plot of effect on postoperative nausea. CI = confidence interval; remi = remifentanil subgroups; nonremi = subgroups without remifentanil administration; I^2^ = heterogeneity; M-H = Mantel–Haenszel.

*Postoperative nausea* Four studies were included in the analysis of nausea [2, 6, 13, 14]. The effect in the remifentanil subgroup suggested that postoperative nausea occurs significant more frequently in the control than in the ADO group (OR = 0.00 [0.00 to 0.07], P < 0.01; however, in the nonremi subgroup, there was no significant difference between the control and ADO group(P = 0.26; Fig 9).

**Fig 10:**
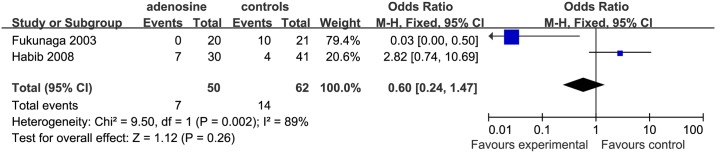
Forest plot of effect on postoperative vomiting. CI = confidence interval; I^2^ = heterogeneity; M-H = Mantel–Haenszel.

*Postoperative vomiting* Two studies included an analysis of postoperative vomiting [2 14]. The effect in the study by Fukunaga et al. (remifentanil subgroup) [14] suggested that this occurred significantly more frequently in the control than in the ADO group (OR = 0.03 [0.00 to 0.50]), but there was no significant difference between controls and the ADO group in the nonremi subgroup (Fig 10).

#### Pruritus

Only 1 study [2] analyzed the occurrence of pruritus; there was no significant difference between the control and ADO group (P = 0.54).

#### Time to first postoperative analgesic requirement

**Fig 11:**
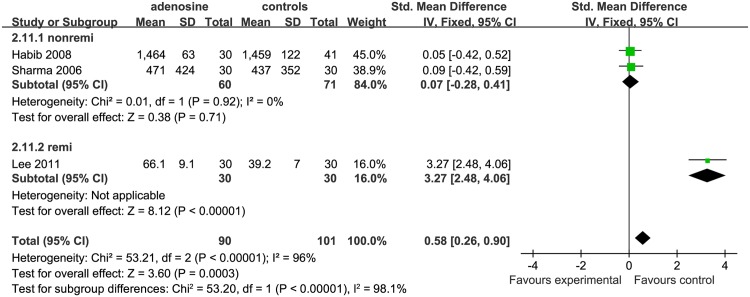
Forest plot of effect on time to first postoperative analgesic requirement (in minutes). CI = confidence interval; I^2^ = heterogeneity; M-H = Mantel–Haenszel; remi = remifentanil subgroups.

Three studies were included in the analysis of the time to first postoperative analgesic requirement (min); there were 2 studies in the saline subgroup [2, 16] and 1 study in the remifentanil subgroup [15]. In the saline subgroup, this outcome was not significantly different in the control compared to the ADO group (SMD = 0.07, 95%CI: −0.28 to 0.41, I^2^ = 0%, P = 0.71); in the remifentanil subgroup, this occurred significantly earlier in the control than in the ADO group (SMD = 1.10, 95%CI: 2.48 to 4.06,P <0.01; Fig 11).

#### Time to hospital discharge

Only the study by Habib et al. [2] observed the effect of ADO in time for hospital discharge. They found no significant difference between the control group with the ADO group(P = 0.78).

#### Effects on cardiovascular system

We assessed the overall effects of ADO on the cardiovascular system, as compared with the control and treatment; several aspects were considered, as follows: systolic blood pressures (SBp), diastolic blood pressures (DBp), heart rate (HR), bradycardia, tachycardia, and transient first -degree atrio-ventricular block (AVB.)

**Fig 12:**
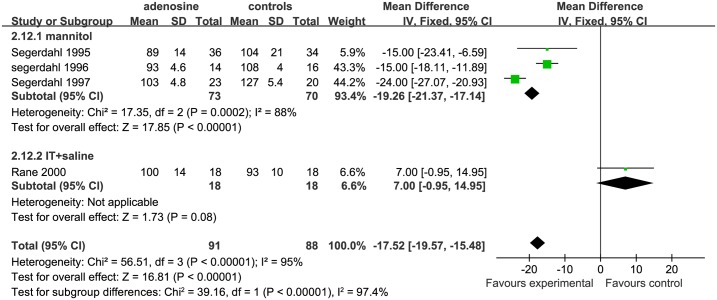
Forest plot of effect on systolic blood pressure in the early surgical period. CI = confidence interval; IV = intravenous injection; IT = intrathecal injection; I^2^ = heterogeneity, early = early surgical period; SBP = systolic blood pressure.

*Systolic and diastolic blood pressures* Both SBP and DBP at different surgical stages were evaluated to determine the effects of ADO on blood pressure. The surgical processes were divided into 3 parts: early surgical period, mid-surgical period, and late surgical period.;. For assessing SBP, 4 studies were eligible;3 studies were includedin mannitol subgroup [6, 7, 9], and only1study[10] was included in the IT+ saline subgroup. The early SBP was significantly lower in the ADO than the control group in the mannitol subgroup (SMD = −19.26, 95%CI:−21.37 to −17.14, I^2^ = 88%, P <0.01), while early SBP was similar between theADOand the control group in the IT+saline subgroup (SMD = 7.00, 95%CI: −0.95 to 14.95, P = 0.08, Fig 12).

**Fig 13:**
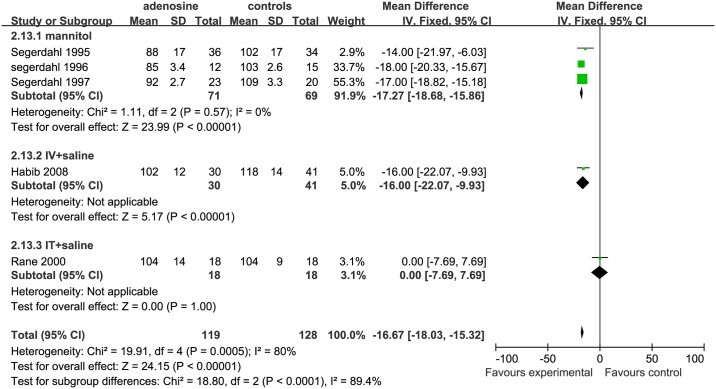
Forest plot of effect on systolic blood pressure in the mid-surgical period. CI = confidence interval; IV = intravenous injection; IT = intrathecal injection; I^2^ = heterogeneity, mid = mid-surgical period; SBP = systolic blood pressure.

For assessing SBP in the mid-surgical period, 5 studies were eligible; 3 studies were included in the mannitol subgroup [6, 7, 9], 1 study[2] in the IV+ saline subgroup, and 1 study[10] in the IT+ saline subgroup. The SBP was significantly lower in the ADO group than in the controls in the mannitol (SMD = −17.27, 95%CI: −18.68 to −15.86, I^2^ = 0%, P <0.01) and IV+ saline(SMD = −16.00, 95%CI: −22.07 to −9.93, P <0.00001) subgroups; while the SBP was similar between ADO treatment and control groups in the IT+saline subgroup (SMD = 0.00, 95%CI:−7.69 to 7.69, P = 1.00; Fig 13).

**Fig 14:**
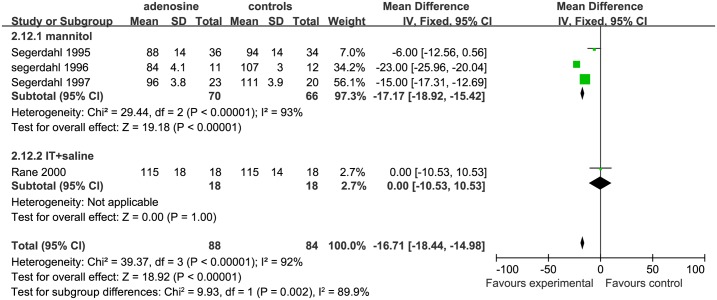
Forest plot of effect on systolic blood pressure in the late surgical period. CI = confidence interval; IV = intravenous injection; IT = intrathecal injection; I^2^ = heterogeneity, late = late surgical period; SBP = systolic blood pressure.

For SBP in the late surgical period, the studies included and the trends of the outcome were almost the same as for the early SBP (Fig 14).

For DBP, only 1 study [2] could be assessed. The study [2] was included in the IV+ saline subgroup. The DBP in the ADO group was significant lower than that in the controls (SMD = −17.00, 95%CI: −21.91 to −12.09, P <0.01).

One study [18] was included in the analysis of hypotension; this study used remifentanil as the control. There was no significant differences between the ADO and control groups (P = 0.15).

**Fig 15:**
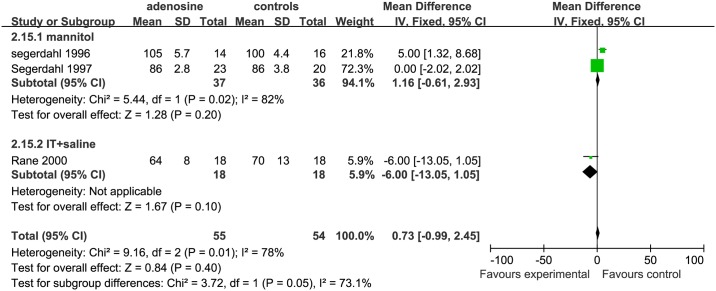
Forest plot of effect on heart rate in the early surgical period. CI = confidence interval; IV = intravenous injection; IT = intrathecal injection; I^2^ = heterogeneity, early = early surgical period; HR = heart rate.

*Heart rate* We also considered the effect of ADO on HR in to 3 parts: early surgical period, mid-surgical period, and late surgical period.separately. For early HR,3 studies were eligible;2 studies were included in the mannitol subgroup [6, 7],and only 1 study[10] was included in the IT+saline subgroup. The early HR in the ADO group was not significantly different from that in the control group in the mannitol (P = 0.20) of IT+ saline subgroups (P = 0.10; Fig 15).

**Fig 16:**
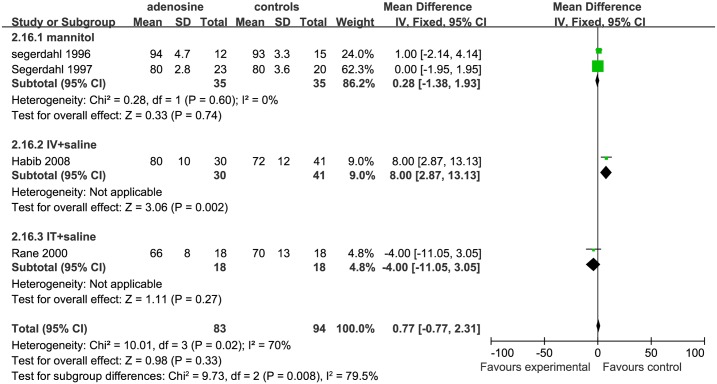
Forest plot of effect on heart rate in the mid-surgical period. CI = confidence interval; IV = intravenous injection; IT = intrathecal injection; I^2^ = heterogeneity, mid = mid-surgical period; HR = heart rate.

For evaluating mid HR, we included 4 studies. 2 studies were included in the mannitol subgroup [6, 7], and 1 study[2] was included in the IV+ saline subgroup and one study[10] in the IT+ saline subgroup. The mid HR in the ADO group was not statistically significantly different from that in the control group in the mannitol(P = 0.74)and IT+ saline subgroups (P = 0.27); however, the mid HR was higher in the ADO group than in the control group in the IV+ saline subgroup (P = 0.002; Fig 16).

**Fig 17:**
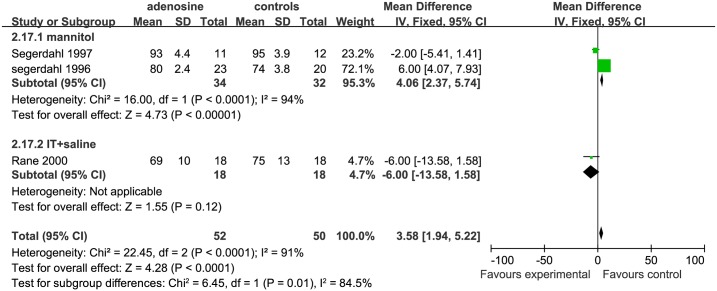
Forest plot of effect on heart rate in the late surgical period. CI = confidence interval; IV = intravenous injection; IT = intrathecal injection; I^2^ = heterogeneity, late = late surgical period; HR = heart rate.

For assessing HR in late surgical period, 3 studies were included: 2 studies were included in the mannitol subgroup[6, 7],while 1 study[10] was included in the IT+ saline subgroup. The HR was significant higher in the ADO group than in the control group in the mannitol subgroup (SMD = 4.06, 95%CI:2.37 to 5.74, P <0.00001). However, the late HR in the IT+ saline subgroup was not significantly different between the treatment and control groups (P = 0.12; Fig 17).

**Fig 18:**
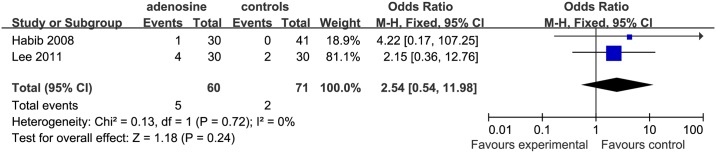
Forest plot of occurrence of bradycardia. CI = confidence interval; I^2^ = heterogeneity; M-H = Mantel–Haenszel.

**Fig 19:**
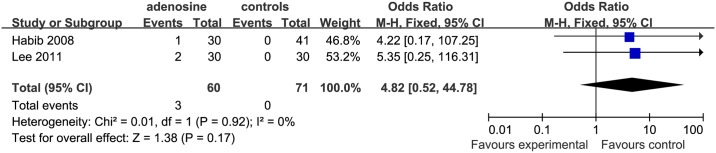
Forest plot of transient first degree atrio-ventricular block. CI = confidence interval; I^2^ = heterogeneity; M-H = Mantel–Haenszel.

For both bradycardia and transient first degree AVB, two studies [2, 15] were eligible for inclusion. 1 study [2] was included in the IV+ saline subgroup, but the study of Lee et al. [15] used remifentanil in the controls. The incidence of bradycardia (P = 0.24) and transient first degree AVB (P = 0.17) was not significantly different in the ADO than in the control groups (Figs 18 and 19).For tachycardia evaluation, 1 study [2] was in it. However, the comparison between ADO and control was not significantly different.

## Discussion

This meta-analysis suggested that ADO administration did not change postoperative VAS/VRS scores by 24 h, irrespective of sex, or whether it was administered by IT or IV, or with mannitol. In comparison with remifentanil, ADO reduced postoperative pain by 24 h, and reduced opioid consumption over the first 48 h; however, in comparison with other non-remifentanil control treatments, ADO had no significant effect on pain score or opioid consumption. Moreover, the time to first analgesic requirement was much earlier in remifentanil-treated than in the ADO group, but ADO treatment did not differ from saline treatment in this respect. The occurrence of PONV was higher in the remifentanil-treated group than in the ADO-treated group. Previous studies [18, 19] had suggested that the occurrence of PONV was associated with an increased usage of opioids. Nevertheless, our study showed no statistically significant difference between ADO and saline treatment. SBP and HR was lower in the ADO treated group than in the control group in the IV+ saline/mannitol subgroups throughout surgery, but in the treatment and control groups did not differ in the IT+ saline subgroup. The occurrence of bradycardia and first degree AVB did not differ statistically significantly between ADO and remifentanil treated groups.

The study of Chiari et al. [20] verified the safety of ADO, and neurological function unaffected in histopathological animal studies. In many studies, ADO has been used as ananalgesic mediator in animal and human experiments [21–25].The present meta-analysis also showed that exogenous ADO relieved postoperative pain by 24h, thereby reducing opioid requirements. These results were in accordance with the studies by Katz et al. and Martins et al. [26, 27] in that endogenous ADO production was found to improve mechanical hyperalgesia. The first postoperative analgesic requirement was about 25 times longer than in the remifentanil subgroup. This effect was thought to be mediated throughout the nervous system by the ADO A1 receptor (A1R) [28]. ADO modulates signal transmission of pain in the periphery and the spinal cord. A1Rs can change the extracellular availability of ADO and subsequently regulate pain transmission. While the study of Sjolund et al. [25] suggested that the pain-reducing effect of ADO is due to a reduction of substance P (SP) in cerebrospinal fluid. It has been suggested that SP facilitates the excitatory amino acid-induced activation of the N-methyl D-aspartate (NMDA) via NK-1 receptor stimulation. The proposed role for SP is supported by the results of behavioral studies in which SP antagonists were used in animal models of peripheral inflammation. The study of Omoigui et al. [29] proposed that the origin of all pain is inflammation and the inflammatory response. However, the effect of ADO was only found in comparison with remifentanil and ADO, but not with saline.

The ORs of PONV in the ADO group was much lower than in the remifentanil subgroups. A study by Murataet al. [30] proposed that ADO serves to counteract further progression of hyperemesis gravidarum. Therefore, ADO seems to have a positive protective action against nausea and vomiting. In this meta-analysis, the effect was only seen in comparison between remifentanil and ADO, but not between saline and ADO.

Moreover, we found that SBP and HR of participants treated with ADO in the IV+ mannitol/saline subgroups were lower than those of the controls throughout the surgery. Thus, ADO administered by IV had marked effects on HR and BP. These effects are probably exerted through theA1, A2A, A2B, and A3 ADO receptor subtypes, which are all expressed in myocardial cells [31 32]. Activation of both the A1Rs and A2Rs is involved in the regulation of HR, whereas A3Rs play a key role in cardioprotection [32–34]. These effects reflected the classic secondary action of ADO.

Thus, ADO had the same impact as saline in terms of analgesia, but reduced heart rate and blood pressure in our meta-analysis. However, the rate of occurrence was not statistically significantly different between ADO and saline groups in terms of bradycardia and transient first- degree AVB. This may be because ADO induces the baroreceptor reflex response to hypotension and directly stimulates the sympathetic nervous system regardless of changes in blood pressure[35].In the study by Takeshi et al. [36], almost 30% of patients demonstrated reduced heart rates after ADO infusion. This could be influenced by smoking, using the β-blockers, higher resting HR, lower ejection fraction, etc., both smoking and β-blockers might destroy the pharmacodynamics of ADO. However, higher resting HR and lower ejection fraction could be associated with the baroreceptor reflex response [35]. The studies included in our meta-analysis did not contain any information on smoking, β-blocker use, or ejection fraction. This should be investigated further in future.

Additionally, ADO is a powerful cardioprotective mediator in ischemic preconditioning. Any factors that are liable to increase ADO accumulation in the heart during myocardial ischemia–reperfusion would reduce myocardial injury [37]. Intracoronary ADO may be an effective therapy for no-reflow in ST-segment elevation myocardial infarction [38]. A meta-analysis by Singh et al. [39] showed that intracoronary ADO administration was well-tolerated and significantly improved electrocardiographic outcomes, with a tendency towards improvement of adverse cardiovascular events, heart failure, and cardiovascular mortality. Safety analysis showed no significant difference in chest pain events (RR 1.26, 95%CI: 0.55 to 2.86; P = 0.58), bradycardia (RR 2.19, 95%CI: 0.24 to 0.38; P = 0.49), ventricular tachycardia (OR 0.61, 95%CI:0.08 to 4.90; P = 0.64), and ventricular fibrillation (RR 0.49, 95%CI: 0.13 to 1.90; P = 0.30), as compared with the placebo group.

Furthermore, ADO activates four receptors (A1R, A2aR, A2bR, A3R) to reduce ischemia–reperfusion injury, as ADO can decrease mechanical obstruction of capillaries by neutrophils. Moreover, it can block the release of vasoconstrictors (e.g.,leukotrienes, platelet activating factor, endothelin) by activated neutrophils and platelets to protect the ischemic myocardium [40]. In the study by Ernens et al. [41], ADO could stimulate increased production of thrombospondin-1 by human macrophages. In rats, chronic ADO administration can increase border-zone vascularization of myocardial infarction lesions, via increase thrombospondin-1 expression. This effect is mediated via the cAMP/PKA pathway and involves A2AR and A2BR [41]. Hence, ADO can improve cardiac function and reduce the area of myocardial infarction in multiple protective ways.

However, our meta-analysis has some limitations. We set out to perform some sensitivity analyses and subgroup analyses to assess the impact of factors on primary outcomes. These analyses may have performance, publication, or reporting bias; and the results should be considered with due caution. The small numbers of included trials was another limitation; we could not apply funnel plots and meta-regression to examine all comparisons and ORs; because these analyses were needed at least 10 studies. The small numbers of included trials caused wide CIs and ORs.

In conclusion, this meta-analysis suggested that ADO has no analgesic effect or prophylactic effect on PONV. However, ADO can reduce SBP and participants’ HR in part during surgery. It did not increase the rate of occurrence of bradycardia and first- degree AVB. This may benefit patients with hypertension, ischemic heart disease (e.g.,coronary heart disease, congestive heart failure) and tachyarrhythmia(e.g.,tachycardia caused by hyperthyroidism, supraventricular tachycardia), to improve cardiac function.

## Supporting information

S1 Checklist PRISMA checklist. (DOC)
